# Ferulic Acid Attenuates TGF-*β*1-Induced Renal Cellular Fibrosis in NRK-52E Cells by Inhibiting Smad/ILK/Snail Pathway

**DOI:** 10.1155/2015/619720

**Published:** 2015-04-08

**Authors:** Ming-gang Wei, Wei Sun, Wei-ming He, Li Ni, Yan-yu Yang

**Affiliations:** ^1^The First Affiliated Hospital of Soochow University, Suzhou, Jiangsu 215006, China; ^2^Jiangsu Province Hospital of Traditional Chinese Medicine, Nanjing, Jiangsu 210029, China

## Abstract

Renal fibrosis is a common cause of renal dysfunction with chronic kidney disease. Central to this process is epithelial-mesenchymal transformation (EMT) of proximal tubular epithelial cells driven by transforming growth factor-*β*1 (TGF-*β*1) signaling. The present study aimed to investigate the effect of Ferulic acid (FA) on EMT of renal proximal tubular epithelial cell line (NRK-52E) induced by TGF-*β*1 and to elucidate its underlying mechanism against EMT related to TGF-*β*1/Smads pathway. The NRK-52E cells were treated for 48 h with TGF-*β*1 (5 ng/mL) in different concentrations of FA (0 to 200 *µ*M). Fibronectin, a mesenchymal marker, was assessed by western blotting. Western blotting was also used to examine the EMT markers (E-cadherin, and *α*-smooth muscle actin (*α*-SMA)), signal transducer (p-Smad2/3), and EMT initiator (Snail). ILK was also assayed by western blotting. The results showed that TGF-*β*1 induced spindle-like morphological transition in NRK-52E cells. Smad2/3 signaling pathway activation, increased fibronectin, *α*-SMA, ILK, and Snail expression, and decreased E-cadherin expression in TGF-*β*1-treated NRK-52E cells. FA efficiently blocked P-Smad2/3 activation and attenuated all these EMT changes induced by TGF-*β*1. These findings suggest that FA may serve as a potential fibrosis antagonist for renal proximal tubule cells by inhibiting EMT process.

## 1. Introduction

Renal fibrosis, characterized by glomerulosclerosis and tubulointerstitial fibrosis, is the final common manifestation of various chronic kidney diseases (CKD), and progressive accumulation and deposition of extracellular matrix (ECM) proteins in the interstitial area are a key feature. The pathogenesis of renal fibrosis is a progressive process that ultimately leads to end-stage renal failure [1–4].

The role of epithelial-to-mesenchymal transition (EMT) of renal tubular epithelial cells has been implicated in accelerating fibrogenesis [5, 6]. Among the many fibrogenic factors that regulate renal fibrotic process, transforming growth factor-*β*1 (TGF-*β*1) has been identified as the most potent mediator and convergent pathway in inducing EMT and renal fibrosis, mainly via the TGF-*β*/Smads signal transduction pathway [7–11]. EMT is a complex process that involves cytoskeletal remodeling, cell-cell and cell-matrix adhesion, and transcriptional regulation, leading to the transition from a polarized epithelial phenotype to an elongated fibroblastoid phenotype and changes in transcriptional regulation to increase cell mobility and invasion [9, 12]. In the process of EMT, a significant decrease in E-cadherin and an increase in *α*-smooth muscle actin (*α*-SMA) expression have been demonstrated for years concomitantly with the increase in Snail protein [5, 13]. Snail is a zinc finger transcriptional factor, functions as a regulator to suppress the expression of adhesion molecules during EMT [14, 15]. The most common biochemical change associated with EMT is the loss of E-cadherin expression regulated by Snail [15]. ILK is well documented as a key intracellular mediator that promotes EMT in different cell systems including tubular epithelial cells [16–19]. Although the molecular details by which ILK induces EMT remain to be elucidated, it seems to be related to its ability to induce key EMT-regulatory gene Snail expression. In concert, the ILK/Snail signaling plays a key role in mediating EMT [18].

Ligusticum chuanxiong has been used in traditional Chinese medicine (TCM) for many years. The clinical effectiveness of* Ligusticum* on chronic renal failure has been evaluated in clinical trials [20]. It can reduce 24-hour urinary protein excretion, ameliorate renal pathological changes, slow down, and even halt the progression of chronic renal failure. Ferulic acid (4-hydroxy-3-methoxycinnamic acid) (FA), an effective component of Ligusticum chuanxiong, is a ubiquitous phenolic acid in the plant kingdom [21]. FA exhibits many physiological functions, including antioxidant, anti-inflammatory, and anticancer activities [22–24]. We previously reported that the Qiguiyishen decoction reduced the accumulation of ECM in the kidneys of rats with Adriamycin-induced nephropathy. FA is one of the most effective components of the Qiguiyishen decoction [25]. However, the antifibrosis effects of FA remain unclear. In this study, we use the in vitro model to investigate the effect of FA in TGF-*β*-induced proximal tubular fibrosis. This study finds that FA plays a role in the regulation of renal fibrosis by inhibiting TGF-*β*-induced renal fibrosis possibly through Smad/ILK/Snail pathway and may lead to possible therapeutic interventions to suppress EMT and renal fibrosis.

## 2. Materials and Methods

### 2.1. Drug and Reagents

Ferulic acid was purchased from Tauto Biotech (Shanghai, China) (purity not less than 98%). Ferulic acid was dissolved in DMSO for its administration. Other chemicals used in this study were of analytical or higher grade.

### 2.2. Cell Culture and Treatment

NRK-52E cells were purchased from the Institute of Biochemistry and Cell Biology (Shanghai, China) and cultured in DMEM/F12 (Gibco, USA) with 10% fetal bovine serum (FBS) (Gibco, USA) in an atmosphere of 5% CO^2^ at 37°C. To determine the effects of FA treatment on the EMT, NRK-52E cells were incubated into 6-well plates with 50–60% confluence, starved for 24 h by incubation with DMEM/F12 containing 0.5% FBS, and then divided into following groups: (1) normal control group incubated in DMEM/F12 containing 0.1% DMSO (i.e., vehicle); (2) TGF-*β*1 group stimulated with recombinant TGF-*β*1 (5 ng/mL; Peprotech); and (3) FA-treated groups stimulated with recombinant TGF-*β* (5 ng/mL) and simultaneously treated with different concentrations of FA (25, 50, 100, and 200 *μ*M). After 48 h, cells were harvested and processed for western blot analysis.

### 2.3. Western Blotting

Western blot assays was used to evaluate the expression of the protein levels. Briefly, cells were lysed in lysis buffer (20 mM Tris, 1 mM EDTA, 1% Triton X-100, 1 mM Na_3_VO_4_, 20 mg/mL Aprotinin, 20 mg/mL Leupeptin, 1 mM DTT, and 1 mM PMSF) and the crude protein lysate was resolved by 12% SDS-PAGE. After protein transfers to a polyvinylidene difluoride (PVDF) membrane and the PVDF membrane was blocked with 5% (w/v) nonfat milk in trisbuffered saline (TBST) for 1 h at 37°C. The blots were probed with a dilution of primary antibody. The primary antibodies used were as follows: antifibronectin (ab23751, Abcam, Cambridge, UK), anti-pSmad2/3 (ab63399, Abcam, Cambridge, UK), anti-Smad2/3 (ab63672, Abcam, Cambridge, UK), anti-E-cadherin (ab133597, Abcam, Cambridge, UK), anti-*α*-SMA (ab5694, Abcam, Cambridge, UK), anti-ILK (ab137912, Abcam, Cambridge, UK), anti-snail (ab180714, Abcam, Cambridge, UK), and *β*-actin (Santa Cruz Biotechnology, Inc.). After hybridization, the blots were washed and hybridized with 1 : 5000 (v/v) dilutions of goat antirabbit IgG, horseradish peroxidase-conjugated secondary antibody (Santa Cruz Biotechnology, Inc.). The signal was generated by adding enhanced Chemiluminescent reagent. *β*-actin was used as an internal control.

### 2.4. Statistics

Data are shown as means ± standard deviation (SD). The statistical difference between groups was determined by the paired Student's *t*-test. A *P* value less than 0.05 was considered significant.

## 3. Results

### 3.1. FA Reversed TGF-*β*1-Induced Morphological Changes in NRK-52E Cells

**Figure 1:**
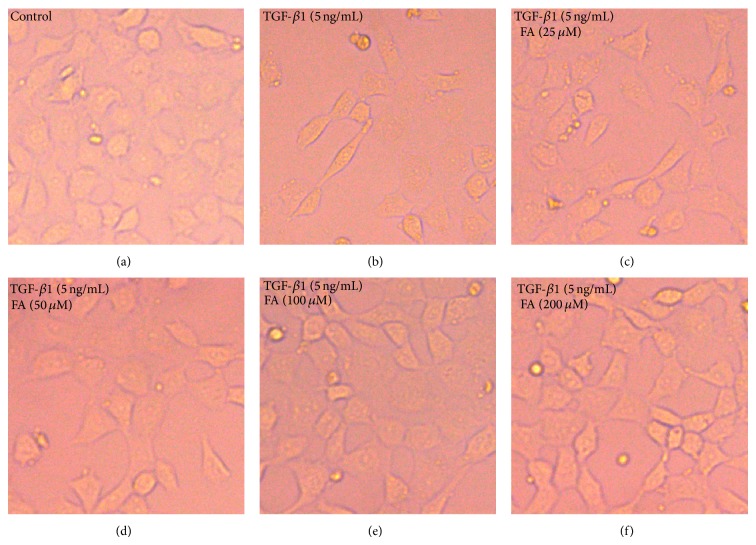
FA reversed TGF-*β*1-induced morphological changes in NRK-52E cells. NRK-52E cells were incubated with 5 ng/mL of TGF-*β*1 for 48 h with different concentrations of FA (0, 25, 50, 100, and 200 *μ*M). (a) Untreated NRK-52E cells showed that a pebble-like shape is clearly observed. (b) TGF-*β*1-treated cells showed a decrease in cell-cell contacts and adopt a more elongated morphological shape. (c), (d), (e), and (f) showed reversed TGF-*β*1-induced morphological changes by different concentrations of FA (magnification of 200x).

Phase-contrast microscopy showed that the cells displayed a pebble-like appearance in the control group. The cells adopted an elongated, fibroblast-like phenotype after the stimulation with recombinant TGF-*β*1. Most importantly, concentrations higher than 50 *μ*M FA attenuated TGF-*β*1-induced the morphological changes mentioned above (Figure 1).

### 3.2. FA Reversed TGF-*β*1-Induced Expression of Fibronectin in NRK-52E Cells

**Figure 2:**
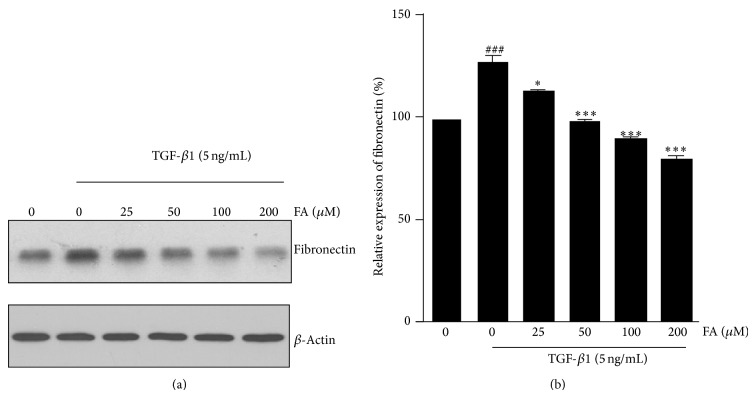
Effects of FA on TGF-*β*1-induced expression of fibronectin in NRK-52E cells. NRK-52E cells were incubated with 5 ng/mL of TGF-*β*1 for 48 h with different concentrations of FA (0, 25, 50, 100, and 200 *μ*M). (a) The intercellular fibronectin was measured by western blotting with *β*-actin used as an internal control. (b) The fibronectin was quantitatively analyzed with Image J software. The data showing mean ± SD. ^#^ *P* < 0.05, ^##^ *P* < 0.01, and ^###^ *P* < 0.005 versus control (0 ng/mL TGF-*β*1). ^∗^ *P* < 0.05, ^∗∗^ *P* < 0.01, and ^∗∗∗^ *P* < 0.005 versus control (0 *μ*M FA in the presence of 5 ng/mL TGF-*β*1).

To evaluate the regulatory effects of FA in TGF-*β*1-induced ECM proteins accumulation, the expression of fibronectin was examined by western blotting. Following the treatment with TGF-*β*1, the expression levels of fibronectin in NRK-52E cells was upregulated compared with the control. FA treatment significantly decreased the production of fibronectin in a dose-dependent manner in NRK-52E cells compared with TGF-*β*1-treated group (Figure 2).

### 3.3. FA Reversed TGF-*β*1-Induced EMT in NRK-52E Cells

**Figure 3:**
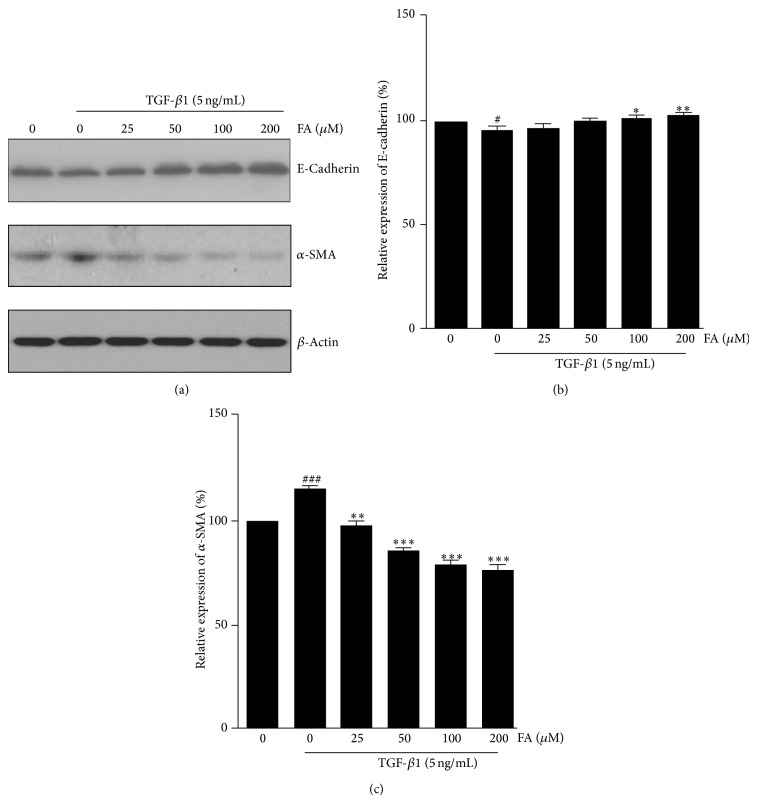
Effects of FA on TGF-*β*1-induced expression of E-cadherin and *α*-SMA in NRK-52E cells. NRK-52E cells were incubated with 5 ng/mL of TGF-*β*1 for 48 h with different concentrations of FA (0, 25, 50, 100, and 200 *μ*M). (a) Expression of E-cadherin and *α*-SMA at the protein level were determined by Western blotting with *β*-actin used as an internal control. (b) and (c) The expression level was quantitatively analyzed with Image J software. The data showing mean ± SD. ^#^ *P* < 0.05, ^##^ *P* < 0.01, and ^###^ *P* < 0.005 versus control (0 ng/mL TGF-*β*1). ^∗^ *P* < 0.05, ^∗∗^ *P* < 0.01, and ^∗∗∗^ *P* < 0.005 versus control (0 *μ*M FA in the presence of 5 ng/mL TGF-*β*1).

To confirm the effect of FA on the EMT in NRK-52E cells, the expression of the epithelial marker E-cadherin, and the mesenchymal marker *α*-SMA were examined by western blotting. As shown in Figure 3, exposure of cells to TGF-*β*1 resulted in a significant reduction in E-cadherin and an increase in *α*-SMA, compared with control. FA significantly prevented TGF-*β*1 stimulated changes of E-cadherin expression at 100 *μ*M and *α*-SMA expression at 25 *μ*M. These results suggest that FA prevents the loss of the epithelial marker Ecadherin and the de novo expression of myofibroblast marker *α*-SMA in renal epithelial cells stimulated by TGF-*β*1.

### 3.4. FA Attenuates TGF-*β*1 Signaling via Smad2/3 Pathway

**Figure 4:**
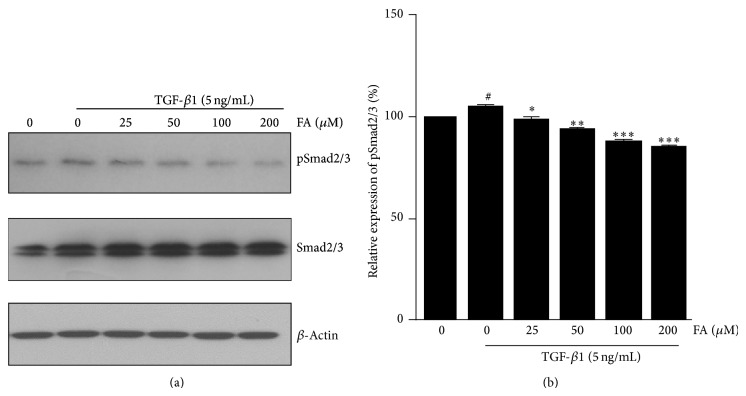
Effects of FA on TGF-*β*1-induced expression of pSmad2/3 in NRK-52E cells. NRK-52E cells were incubated with 5 ng/mL of TGF-*β*1 for 48 h with different concentrations of FA (0, 25, 50, 100, and 200 *μ*M). (a) Expression of Smad2/3 and pSmad2/3 at the protein level was determined by western blotting with *β*-actin used as an internal control. (b) The pSmad2/3 was quantitatively analyzed with Image J software. The data showing mean ± SD. ^#^ *P* < 0.05, ^##^ *P* < 0.01, and ^###^ *P* < 0.005 versus control (0 ng/mL TGF-*β*1). ^∗^ *P* < 0.05, ^∗∗^ *P* < 0.01, and ^∗∗∗^ *P* < 0.005 versus control (0 *μ*M FA in the presence of 5 ng/mL TGF-*β*1).

Activation of smad2/3 by phosphorylation is the central process of the EMT response to TGF-*β*1 [26]. Exposure to TGF-*β*1 resulted in significantly increased Smad2/3 phosphorylation, compared with control. FA treatment significantly decreased the phosphorylation of Smad2/3 in a dose-dependent manner in NRK-52E cells compared with TGF-*β*1-treated group. Concentrations higher than 25 *μ*M significantly blocked Smad2/3 phosphorylation protein expression (Figure 4).

### 3.5. Effect of FA on the ILK and Snail Expression in NRK-52E Cells

**Figure 5:**
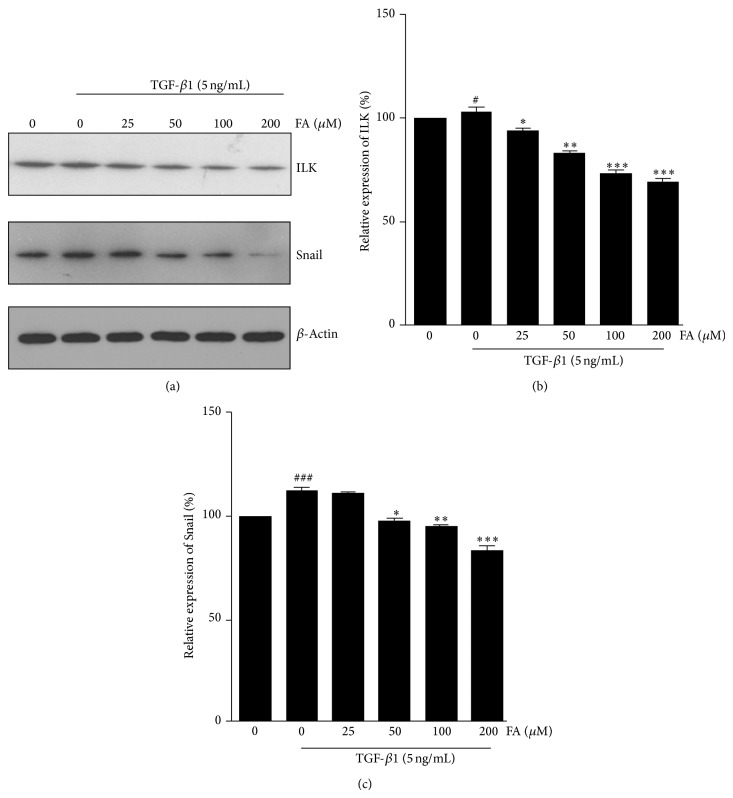
Effects of FA on TGF-*β*1-induced expression of ILK and Snail in NRK-52E cells. NRK-52E cells were incubated with 5 ng/mL of TGF-*β*1 for 48 h with different concentrations of FA (0, 25, 50, 100, and 200 *μ*M). (a) Expression of ILK and Snail at the protein level were determined by western blotting with *β*-actin used as an internal control. (b) and (c) The expression level was quantitatively analyzed with Image J software. The data showing mean ± SD. ^#^ *P* < 0.05, ^##^ *P* < 0.01, and ^###^ *P* < 0.005 versus control (0 ng/mL TGF-*β*1). ^∗^ *P* < 0.05, ^∗∗^ *P* < 0.01, and ^∗∗∗^ *P* < 0.005 versus control (0 *μ*M FA in the presence of 5 ng/mL TGF-*β*1).

ILK has been shown to be a key intracellular mediator that controls EMT in tubular epithelial cells by inducing key EMT-regulatory gene Snail expression [18]. Because of the association of Snail with EMT, we then investigated the expression of ILK and Snail in NRK-52E cells to underlying mechanism that FA inhibited EMT. As shown in Figure 5, the expression of ILK and Snail in NRK-52E cells was increased in response to TGF-*β*1 treatment, and the FA markedly decreased TGF-*β*1-induced ILK and Snail protein expression.

## 4. Discussion

Renal tubulointerstitial fibrosis is the final consequence of chronic kidney disease which leads to the destruction of the kidney and end-stage renal failure [27, 28]. Renal fibrosis is associated with tubular epithelial cells transition to mesenchymal cells via a process known as EMT. EMT is an important process in the pathogenesis of tubulointerstitial fibrosis and involves a loss of epithelial cell characteristics and an increase of mesenchymal cell markers stimulated by various profibrotic cytokines. Therefore, blocking renal EMT may prevent renal fibrosis. TGF-*β*1 is a well-known profibrotic cytokine in several renal diseases and plays a critical role in the renal EMT process. In this study, we used an in vitro model and NRK-52E cells stimulated by TGF-*β*1 to address whether FA attenuates renal interstitial fibrosis in vitro and to investigate the underlying mechanisms.

Here we show that exposure to TGF-*β*1 for 48 h in NRK-52E cells induce morphologic change and an upregulation of ECM protein, fibronectin. TGF-*β*1 also increases *α*-SMA and decreases E-cadherin expression in NRK-52E cells. However, FA significantly reverses all of above changes in vitro. These results suggest that FA prevents TGF-*β*1-mediated renal EMT in vitro. The mechanism by which FA inhibits TGF-*β*1-induced EMT remains unknown. It is more likely that multiple pathways are involved in the inhibitory effect of FA, among which Smad2/3 activation, ILK, and Snail expression may be the major events during the process. The exact mechanism for the effects of FA on TGF-*β*1-induced EMT needs to be further elucidated.

TGF-*β*1 and its downstream signaling pathway were shown to play a critical role in activating cellular pathological mechanisms in renal tubulointerstitial fibrosis through the induction of interstitial cell activation and the expression of several profibrotic genes. It has been demonstrated that activation of TGF-*β*1 signaling triggers a dramatic induction of Smad2/3 phosphorylation. In this study, we investigated whether it could also block TGF-*β*1-induced Smad2/3 activation in NRK-52E cells. Our results showed that FA inhibited the p-Smad2/3 activation induced by TGF-*β*1 in NRK-52E cells. We also further studied the effects of FA on Snail expression, which was the important downstream mediator of TGF-*β*1/Smads signaling pathway. Our results showed that treatment of FA in NRK-52E cells attenuated TGF-*β*1-induced upregulation of Snail expression. The present study demonstrated that FA could block TGF-*β*1-induced EMT probably by inhibition of p-Smad2/3 activation and Snail expression in NRK-52E cells.

Previous studies have shown that ILK is a key intracellular mediator of TGF-*β*1-induced EMT. TGF-*β*1 regulation of EMT has been suggested to be dependent on integrin-linked kinase (ILK) function during renal fibrosis [18]. TGF-*β*1 induced ILK expression in renal tubular epithelial cells is dependent on intracellular Smad signaling. ILK is a widely expressed serine/threonine protein kinase, which localizes to focal adhesion plaques and centrosomes. ILK has a fundamental role in the regulation of cell survival, proliferation, and migration by connecting the cytoplasmic domains of *β*-integrins to the actin cytoskeleton, mediating integrin signaling in diverse cell types. Together, ILK is a key intracellular mediator of TGF-*β*1-induced EMT. We further test the effect of FA on the expression level of ILK. As shown in Figure 5, treatment of FA in NRK-52E cells attenuated TGF-*β*1-induced upregulation of ILK expression. The above observation demonstrated that FA attenuates renal EMT and ECM protein expression in NRK-52E cells induced by TGF-*β*1. The possible mechanism involves the suppression of the TGF-*β*1/Smad2/3 pathway and the subsequent inhibition of ILK and snail expression and inhibition of EMT processes by restoring the expression of E-cadherin in renal proximal tubules.

## 5. Conclusion

In conclusion, our data present, for the first time, the important information that FA, as an herbal compound, inhibits TGF-*β*1-induced EMT in NRK-52E cells. These findings may pave the way for an effective therapy for tubulointerstitial fibrosis.
